# Multiple Marginal Tissue Recession Treated with a Simplified Lateral Sliding Flap Technique

**DOI:** 10.1155/2014/432960

**Published:** 2014-10-21

**Authors:** Fernando Salimon Ribeiro, Juliana Aparecida Najarro Dearo de Morais-Camillo, José Marcos Alves Fernandes, Juliana Rico Pires, Elizangela Partata Zuza, Ana Emília Farias Pontes

**Affiliations:** ^1^Masters of Science Program, Educational Foundation of Barretos, UNIFEB, 14783-226 Barretos, SP, Brazil; ^2^Private Practice, 37958-000 Monte Santo de Minas, MG, Brazil; ^3^School of Dentistry, Federal University of Goiás, 74605-220 Goiânia, GO, Brazil

## Abstract

Marginal tissue recession is a common esthetic problem that is usually accompanied by dentin sensitivity, and patients frequently report a fear of dental loss. Lateral sliding flaps have been used for localized recession, but they are rarely used for multiple recessions. The aim of this paper was to report a case of coverage of multiple marginal tissue recessions by means of a lateral sliding flap associated with a connective tissue graft. This was a modification of Nelson's technique, which was originally described as the combination of the double papilla technique, lateral sliding flap, and connective tissue graft. In the present case, double papilla was not performed, rendering the maneuver less complicated. After surgery on teeth #23 to #25, total root coverage, decreased dentin sensitivity, and increased keratinized tissue band and gingival thickness were achieved. In the present case, modified Nelson technique proved to be a more simple procedure for the treatment of multiple recessions in one session, resulting in adequate healing, predictable root coverage, and, more importantly, esthetic and functional success.

## 1. Introduction

Increasing interest in dental esthetics has significantly influenced treatment planning over the past few years. Subsequently, new dental materials and techniques have been developed in order to provide predictable esthetic outcomes. Among esthetic buccal disorders, marginal tissue recession—due to the loss of marginal gingival tissues, periodontal connective tissue fibers, tooth cementum, and alveolar bone—has garnered particular attention [1].

Marginal tissue recessions are multifactorial and are caused by traumatic tooth-brushing techniques and periodontal disease, not to mention occlusal trauma, the presence of partially removable prosthesis clamps, improper restorations, frenal pull, tooth malposition, extraction, or inadequate incision that can act individually or in combination to initiate the problem [2, 3]. As a consequence, impaired esthetic appearance, dentin sensitivity, and root caries may occur, and patients often report fear of dental loss [2].

Surgical periodontal techniques include free gingival grafts, connective tissue grafts, coronally positioned flaps, and laterally sliding flaps [4–8]. These techniques can be performed alone or in combination to perform root coverage of multiple recessions during the same procedure with predictable outcomes [9].

For lateral sliding flaps, keratinized tissue from the interdental area is dislocated and positioned over the exposed root, as described by Grupe and Warren Jr. [10] in 1956. This is considered one of most predictable techniques, likely due to the maintenance of a pedicle, which provides a double blood supply at the recipient site, resulting in good color and texture blend [11]. Although mostly used to treat isolated dental recessions, Nelson [5] described a technique for multiple root coverage, combining lateral sliding flap, double papilla, and connective tissue graft.

The aim of this paper was to report a case of coverage of multiple marginal tissue recessions by means of a lateral sliding flap associated with a connective tissue graft. This modified, simplified Nelson technique rendered double papilla intervention unnecessary.

## 2. Case Report

A 40-year-old man reported to the Periodontics Clinic of the Araraquara School of Dentistry at the State University of São Paulo (UNESP) with dentin sensitivity in the left upper posterior region. Written consent was obtained, and this report was conducted in full accordance with ethical principles, including the World Medical Association Declaration of Helsinki.

Upon clinical examination, the presence of marginal tissue recessions (Miller Class II) was detected on the buccal aspect of teeth #23, #24, and #25. Traumatic tooth brushing was defined as the primary etiology. In these teeth, the recessions measured 5 mm, and probing depths greater than 3 mm were not identified; plaque index and sulcular bleeding on probing were less than 20%.

The treatment plan included oral hygiene instruction to adjust the pattern of tooth brushing, and periodontal plastic surgery was scheduled 4 weeks later, by means of connective tissue graft associated with a lateral sliding flap.

**Figure 1:**
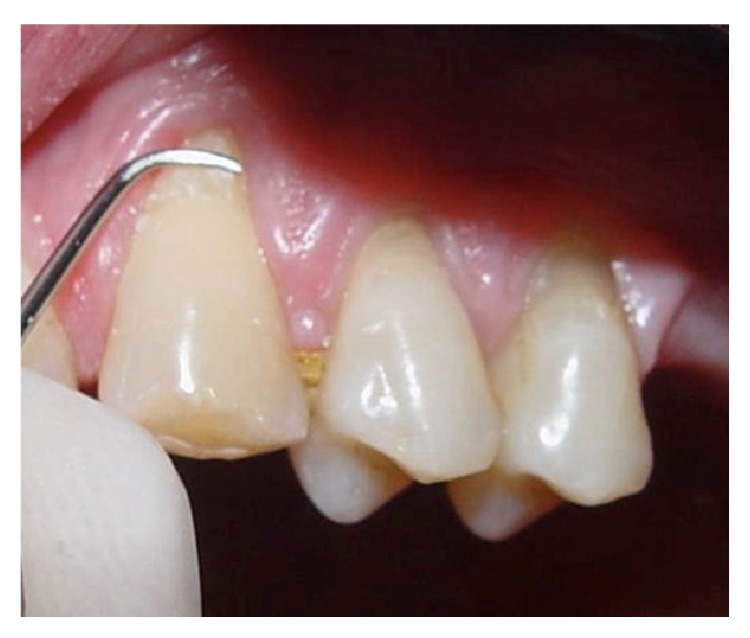
Multiple marginal tissue recessions on teeth #23, #24, and #25 were scaled and planed prior to the surgical procedure.

On the day of the surgery, extraoral and intraoral antisepsis were performed with a 0.12% chlorhexidine digluconate solution, and local anesthesia was performed with mepivacaine 2% HCl with norepinephrine (1 : 100,000). Next, scaling and planning of the exposed roots were performed using Gracey 7-8 curettes, with the aim of removing infected cementum and preparing the roots for coverage (Figure 1).

**Figure 2:**
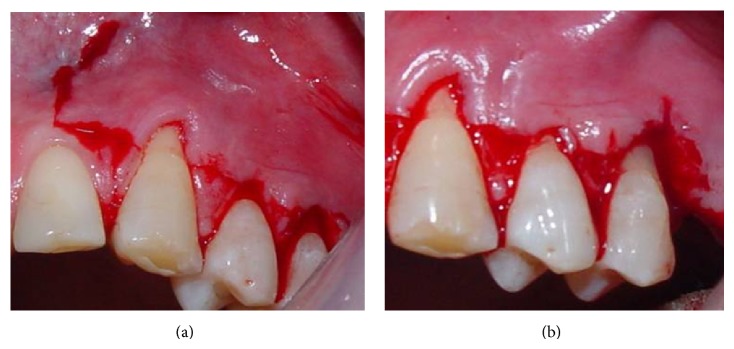
Vertical and horizontal incisions were performed from the mesial portion of (a) tooth #23 up to (b) tooth #25.

**Figure 3:**
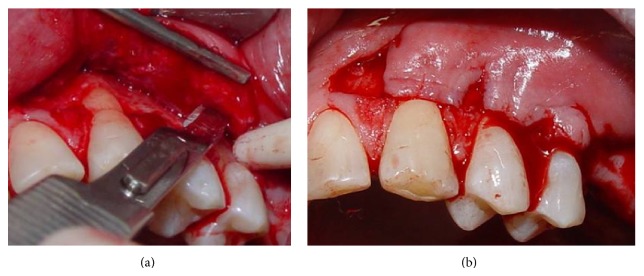
(a) A linear incision was performed on the periosteum at the base of the flap, (b) to allow positioning of the flap without tension before suturing.

**Figure 4:**
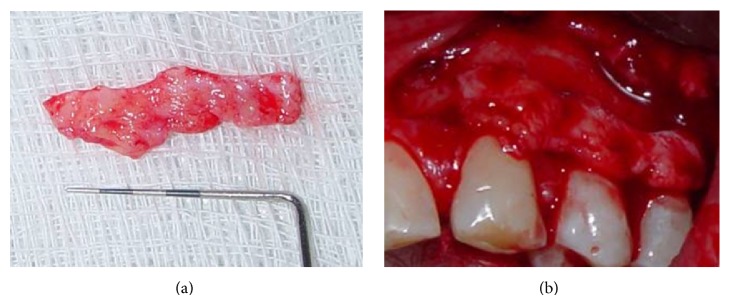
(a) Connective tissue graft was harvested from the palate and (b) positioned over the recipient site.

A sulcular incision was performed from teeth #23 to #25, connected by horizontal incisions on the papilla base. Then, vertical incisions in the mesial surface of tooth #23 and the distal surface of tooth #25 were performed (Figure 2). In the apical portion of the vertical incision in the mesial surface of tooth #23, a cutback incision was performed in order to provide mobility to the flap. Afterwards, a double flap was raised with partial thickness on the mesial portion of tooth #23 and full thickness in the remaining portions, as proposed by Espinel and Caffesse [12]. A linear incision was performed on the periosteum at the base of the flap to allow for positioning of the flap without tension (as shown in Figure 3), before suturing. A connective tissue graft was obtained from the palate (Figure 4(a)) using the linear technique proposed by Paolantonio [8]. Next, the connective tissue graft was positioned over the recipient site (Figure 4(b)).

Interdental and lateral interrupted, absorbable suture Vicryl 6.0 (Ethicon Inc., Johnson & Johnson Company, São Bernardo do Campo, SP, Brazil) were used to stabilize the flap. This flap was then laterally advanced to completely cover the connective tissue graft and was stabilized with interrupted mattress 4-0 silk sutures (Ethicon Inc.). In order to stabilize the clot, the donor site was sutured with interrupted “X” sutures.

**Figure 5:**
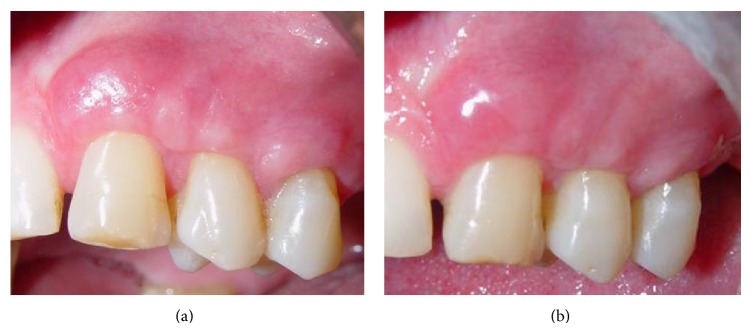
Clinical aspects of the site (a) 30 days and (b) 8 months after surgery.

Ibuprofen 400 mg was prescribed every 6 hours for 3 days, and 0.12% chlorhexidine digluconate solution was prescribed twice daily for 2 weeks. Follow-up control sessions were performed weekly during the first month (Figure 5(a)). Seven days after surgery, good healing was noticed on the host and donor sites. Eight months after surgery (Figure 5(b)), esthetic clinical outcome was observed, with complete root coverage and formation of an adequate band of keratinized tissue. The patient was fully satisfied with the functional and esthetic results and reported a significant decrease in dentin sensitivity.

## 3. Discussion

The treatment of marginal tissue recession has been a therapeutic challenge, mainly when dealing with multiple teeth. The combination of techniques, however, allows for the treatment of adjacent recessions in one session [2, 12, 13].

Lateral sliding flap was chosen in the present case because there was enough volume of keratinized tissue in the interdental areas. Moreover, the combination of lateral sliding flap and connective tissue graft offered the advantages of both techniques. The first technique creates a zone of keratinized gingival tissue and the second technique results in color blending, increased gingival thickness, reduced morbidity in the donor site, and a high degree of root coverage [8, 13, 14]. Graft survival using this technique is high because full thickness lateral sliding flaps offer additional vascularization of the graft [4, 6, 12, 15–17]. In addition, this type of flap allows keratinized tissue from the papilla to be placed over the area of the recession, increasing the chance of keratinized tissue formation in the surgical area [18–20].

According to Smukler [18], Guinard and Caffesse [19], and Romanos et al. [21], the success of lateral sliding flap when used alone is around 70% root coverage. However, if lateral sliding flap is associated with connective tissue graft, root coverage reaches around 95%.

The connective tissue graft is considered the gold standard for root coverage procedures [9]. Studies have shown a mean recession defect coverage of 96% [8, 13, 14, 20]. This may be attributed to the increase in gingival thickness due to the connective tissue graft. On the other hand, one disadvantage, especially with respect to multiple recessions, is that the patient can experience pain in the donor area. This is related to the surgical technique used and the volume of the removed graft. In the present case, a linear technique that maintains tissue with blood irrigation and decreases the risk of necrosis was used to minimize this disadvantage [7].

During the postoperative period, our patient reported a decrease in dentin sensitivity. Harris [6] also reported a decrease in dentin sensitivity in 68 of the 100 cases studied. Thus, dentin sensitivity in recession areas can be an indication for the use of surgical procedures [3, 4, 6, 8, 12, 15–17].

Thus, in the present case, the modified Nelson technique resulted in the treatment of multiple recessions in a single procedure, resulting in adequate healing, predictable root coverage, and, more importantly, esthetic and functional success.
